# Herd clustering strategies and corresponding genetic evaluations based on social–ecological characteristics for a local endangered cattle breed

**DOI:** 10.5194/aab-64-187-2021

**Published:** 2021-05-26

**Authors:** Jonas Herold, Kerstin Brügemann, Sven König

**Affiliations:** Institute of Animal Breeding and Genetics, University of Giessen, 35390 Giessen, Germany

## Abstract

The accuracy of breeding values strongly depends on the population and herd structure, i.e., the number of animals considered in genetic evaluations and the size of contemporary groups (CGs). Local breeds are usually kept in small-sized family farms under alternative husbandry conditions. For such herd structure, consideration of classical herd or herd-test-day effects in CG modeling approaches implies only a few records per effect level. In consequence, the present study aimed on methodological evaluations of different herd clustering strategies, considering social–ecological and herd characteristics. In this regard, we considered 19 herds keeping cows from the small local population of German Black Pied cattle (*Deutsches Schwarzbuntes Niederungsrind*; DSN), 10 herds keeping Holstein Friesian (HF) cows and one mixed herd with HF and DSN cows. Herds were characterized for 106 variables, reflecting farm conditions, husbandry practices, feeding regime, herd management, herd fertility status, herd health status and breeding strategies as well as social–ecological descriptors. The variables were input data for different clustering approaches including agglomerative hierarchical clustering (AHC), partition around medoids (PAM), fuzzy clustering (FZC) and a clustering of variables combined with agglomerative hierarchical clustering (CoVAHC). The evaluation criterion was the average silhouette width (ASW), suggesting a CoVAHC application and consideration of four herd clusters (HCs) for herd allocation (ASW of 0.510). HC1 comprised the larger, half organic and half conventional DSN family farms, which generate their main income from milk production. HC2 consisted of small organic DSN family farms where cows are kept in tie stables. HC3 included the DSN sub-population from former East Germany, reflecting the large-scale farm types. The specialized HF herds were well separated and allocated to HC4. Generalized linear mixed models with appropriate link functions were applied to compare test-day and female fertility traits of 5538 cows (2341 DSN and 3197 HF) from the first three lactations among the four HCs. Least squares means for milk, fat and protein yield (Mkg, Fkg and Pkg) significantly differed between HC. The significant differences among the four HCs clearly indicate the influence of varying herd conditions on cow traits. The similarities of herds within HC suggested the application of HCs in statistical models for genetic evaluations for DSN. In this regard, we found an increase of accuracies of estimated breeding values of cows and sires and of heritabilities for milk yield when applying models with herd-cluster-test-day or herd-cluster-test-month effects compared to classical herd-test-day models. The identified increase for the number of cows and cow records in CG due to HC effects may be the major explanation for the identified superiority.

## 1 Introduction

Local cattle breeds contribute to genetic diversity and may carry favorable alleles with relevance for future production systems and market requirements, justifying efforts for the implementation of preservation programs (Ajmone-Marsan et al., 2010; Toro et al., 2011). Numerous studies (Toro et al., 2011; Fernández et al., 2011; Biscarini et al., 2015; Mastrangelo et al., 2016; Cervantes et al., 2016) focused on strategies to maintain genetic variability in endangered breeds and especially focused on the minimization of inbreeding and genetic drift. A major feature of local endangered cattle breeds is their ability for adaptation to harsh environments (Fernández et al., 2011; Cervantes et al., 2016). In consequence, local cattle breeds are mostly kept in alternative production systems, reflecting a broad pattern of challenges such as limited food resources or climatic stress (Halli et al., 2020). In Germany, the local dual-purpose cattle breed German Black Pied cattle (*Deutsches Schwarzbuntes Niederungsrind*; DSN) is used for dairy cattle farming mainly in small-sized organic herds reflecting harsh environments. The DSN breeding goal considers both milk and meat production, and it focuses on genetic improvements for longevity and female fertility. The current DSN population size comprises about 3500 registered cows (Jaeger et al., 2018).

Accurate genetic evaluations in DSN are imperative, because several German breeding organizations sell semen from DSN sires worldwide. DSN and Holstein Friesian (HF) cattle are considered simultaneously in genetic evaluations, but their genetic connectedness is quite low, implying biased estimated breeding values (EBVs) when ignoring further genetic model improvements. In such context, Jaeger et al. (2019) suggested improved genetic evaluations for DSN through a widened population size, i.e., considering DSN cows from the Netherlands and from Poland.

In addition to the small DSN population size, accuracy of selection and genetic evaluations is hampered due to the small-sized herd structures. Classically, genetic evaluation models consider a herd effect, because the herd usually represents same management, feeding or husbandry conditions for all cows from the same herd. However, genetic (co)variance components and EBVs might be biased when creating specific management groups within herds or when applying preferential treatment for specific cow groups (König et al., 2005). Accordingly, Kennedy and Trus (1993) stretched the topic of contemporary groups (CGs) in genetic evaluations, which should represent the microenvironment as detailed as possible. The influence of the herd effect when modeling CGs is not constant throughout the year, implying consideration of a further time-dependent explanatory variable, i.e., via herd-year-season or herd-test-day modeling (Emmerling, 2000). However, such modeling approaches might be problematic in the case of small herd sizes, implying only a few cow records per CG, with detrimental impact on selection accuracy (Strabel et al., 2005; Pereira et al., 2018). Furthermore, human–animal relationships reflecting social characteristics determine herd effects. Social characteristics plus classical environmental conditions (e.g., climate, feeding resources) were considered when defining social–ecological systems for livestock classifications (Martin-Collado et al., 2014). In small-sized DSN family farms, Ebinghaus (2018) identified impact of the individual farm management and human–animal interactions on variations of disease incidences and production levels across herds (Ebinghaus, 2018).

Alternative modeling strategies to create CG were introduced by Strabel et al. (2005) and Vasconcelos et al. (2008). They grouped herds according to herd size or average milk yield per herd. Grouping herds according to herd, environmental or social characteristics points to the application of herd clustering strategies. Table 1 gives an overview of the clustering methods as applied in cattle populations. Key factors to allocate herds to herd clusters (HCs) were the overall production system (conventional or organic), herd size, breed, country or region (Blanco-Penedo et al., 2019; Ivemeyer et al., 2017). Tremblay et al. (2016) recommended that clustering approaches should consider variables reflecting herd particularities.

**Table 1:** Overview for applied herd clustering approaches.

Author	Method${}^{*}$	Number of	Number of variables		Number of
		herds	Collected	Used for grouping	herd clusters
Blanco-Penedo et al. (2019)	AHC	192	114	16	3
Ivemeyer et al. (2017)	Two-step cluster analysis	204	90	4	4
Brotzman et al. (2015)	AHC	557	22	16	6
Köbrich et al. (2003)	AHC	67		7	5
	AHC	72	40	8	5
Savoia et al. (2019)	AHC	115		4	6
Guiomar et al. (2018)	PAM	916	22	5	8
Gorgulu (2010)	FZC	136	7	7	4
Kuentz-Simonet et al. (2015)	CoVAHC	544	67	9	7
Tremblay et al. (2016)	Mixed latent-class model-based clustering	529	20	18	6
Salasya and Stoorvogel (2010)	FZC	296	11	11	3
Weigel and Rekaya (1999)	k-means	45.936	13	9	5

${}^{*}$ AHC is agglomerative hierarchical clustering, PAM is the partition around medoids, FZC is fuzzy clustering, CoVAHC is the clustering of variables combined with agglomerative hierarchical clustering, and k-means clustering is the nearest centroid method.

The objective of this study was to evaluate different HC strategies including a sample of DSN herds and some HF herds, with the aim of defining appropriate CGs in genetic evaluations. Created clusters will be described based on descriptive statistics for HC variables, as well as on detailed analyses for cow traits in respective HCs using mixed model applications. In a last step, we evaluated the impact of HC modeling on genetic parameter estimates and on accuracies of genetic evaluations.

## 2 Materials and methods

### 2.1 Herd characterization

Herd data were collected via face-to-face interviews and farm characterizations between September 2017 and March 2018, considering 19 DSN herds, 10 HF herds and one mixed herd keeping both breeds. The 19 DSN herds reflected “pure” DSN herds with DSN gene percentages larger than 87.5 %, considering the algorithm for gene percentage calculations as developed by Jaeger et al. (2018). Also the DSN cows in the mixed herd were pure DSN. The HF herds were chosen to consider a genetically related breed, but with opposite breeding goals, production levels and farming systems. The participating herds were located in three major geographic regions from Germany: (1) intensive grazing systems on coastal marshlands, (2) large-scale farms (indoor system) in one region of former East Germany and (3) small-scale family farms in the middle of Germany (semi-intensive grazing systems with maximal 5 h grazing per day). The altitudes of farms in the three regions were 14.68, 84.00 and 200.47 m, respectively, and the latitudes were 53${}^{\circ }$28${}^{\prime }$, 51${}^{\circ }$67${}^{\prime }$ and 51${}^{\circ }$12${}^{\prime }$, respectively. The median herd size for all herds comprised 95 milking cows, ranging from 3 to 800 cows (median 35.5) for DSN and from 60 to 780 cows (median 156.5) for HF.

The farm visits for structured interviews and herd characterizations comprised 45 to 90 min per farm. The survey and visual herd observations included quantitative and qualitative information with regard to general herd characteristics, the feeding regime, the housing system, the husbandry practices, the herd and pasture management, herd fertility and health status as well as the management of calves, heifers and dry cows. Social components addressed, e.g., the herd manager's education, the expenditure of time used for dairy cattle farming, the family status, the number and age of the children and the number of farm employees. In total, the herd characterization comprised 117 variables (26 quantitative and 91 qualitative variables) as indicated in the Supplement (Table S1). Answers were possible via multiple choice but also included open questions and required specific numeric values in some cases (Supplement Table S1). Quantitative variables were scaled by a z transformation, implying a mean of 0 and a variance of 1 (Gagaoua et al., 2018). The data collection was carried out by the same interviewer, so that a misinterpretation of questions by the farmer can be excluded.

### 2.2 Herd clustering approaches

The previously applied HC approaches (as summarized in Table 1) focused on one specific method. In the present study, we compared four different HC methods, especially from the perspective of HC consideration in genetic evaluations. The following four different HC methods were applied: (i) agglomerative hierarchical clustering (AHC), (ii) partition around medoids (PAM), (iii) fuzzy clustering (FZC) and (iv) a clustering of variables combined with agglomerative hierarchical clustering (CoVAHC). All clustering analyses were conducted in R version 4.0.2 (R Core Team, 2020) and applying the packages “cluster” (Maechler et al., 2018) and “ClustOfVar” (Chavent et al., 2017). According to Pimenta et al. (2017), herd variables indicating limited variation or strong correlations with other variables, were deleted. After herd variable editing, 106 variables (23 quantitative and 83 qualitative variables) remained for the ongoing cluster analyses. Based on the mixed data types (nominal, ordinal, (a)symmetric binary, metric), the Gower distance (Gower, 1971) modified by Struyf et al. (1996), was used to calculate the dissimilarity matrix. The overall average silhouette width (ASW) as defined by Rousseeuw (1987) was used to evaluate the clustering approaches and to identify the optimal number of HCs. The silhouette width ranges between -1 and 1, whereby a good cluster separation is characterized by a high intra-homogeneity and inter-heterogeneity with values close to 1 (Rousseeuw, 1987; Lletí et al., 2004; Gagaoua et al., 2018). For all clustering approaches, we calculated the ASW for each number of HCs (evaluated range: 2–10; according to the studies as listed in Table 1).

*Agglomerative hierarchical clustering (AHC)*: The aim of this algorithm is to identify a hierarchical clustering of elements based on similarities or dissimilarities. Initially, each element is considered as a single cluster. Afterwards, these clusters are merged stepwise until the complete data set becomes a cluster (Struyf et al., 1996; Köbrich et al., 2003). The approach recommended by Ward (1963) was used to create homogeneous clusters by fusion. This approach is based on a classical sum-square criterion and produces clusters that minimize variation within the group at each merging step (Murtagh and Legendre, 2014). The dissimilarities can be used to visualize each merging steps in a dendrogram with horizontal lines indicating a combination of herds or HCs.

*Partition around medoids (PAM)*: This method is an upgrade of the popular k-means algorithm, which is fast, efficient and simple. k-means clustering only handles numeric values, but PAM also works with ordinarily scaled variables (Maione et al., 2019). PAM is more robust to outliers than k-means clustering, because it minimizes the sum of non-squared dissimilarities instead of the sum of squared Euclidean distances (Kaufman and Rousseeuw, 1990; Struyf et al., 1996). PAM searches for k medoids (the representative elements) within the data set (Kaufman and Rousseeuw, 1990) and minimizes the total dissimilarity of each element to its nearest medoid.

*Fuzzy clustering (FZC)*: In contrast to AHC and PAM, where each element belongs exactly to one cluster, FZC is a so-called soft clustering algorithm. This means that an element can be assigned to varying clusters. Each element receives a membership value that indicates how strongly the element belongs to any cluster (Struyf et al., 1996; Salasya and Stoorvogel, 2010). The membership exponent $r\left[1\to \infty \right]$ describes the degree of fuzziness, where r=1 is comparable to a strict clustering such as AHC or PAM, and r=∞ is the highest degree of fuzziness (Salasya and Stoorvogel, 2010).

We varied r in the range from 1.0 to 4.0. The best HC differentiation was realized for r=1.1. Hence, all of the following presented results are based on r=1.1.

*Clustering of variables combined with agglomerative hierarchical clustering (CoVAHC)*: CoVAHC is a combination of “clustering of variables” (herd information) followed by an AHC (see above) of the resulting “synthetic variables”. The aim of the “clustering of variables” (CoV) is to find a partition (one that contains similar information) in a mixed data set, in which variables are arranged in homogeneous groups by means of a hierarchical algorithm. This algorithm forms a set of p partitions of variables according to the following scheme:

1. Start partition: each variable is one start cluster.
2. Two clusters will be merged to a new partition when the dissimilarity is the smallest, so that the loss of homogeneity of the new cluster is minimal. This merging step is repeated until each variable is grouped with another variable or partition.
3. End partition: all start clusters form one complete cluster.

The CoV focuses on two aspects. The first is merging closely related variables by grouping them into partitions that maximize the homogeneity criterion, which is defined by the sum of squared Pearson correlations for quantitative variables and correlation ratios for qualitative variables. If all quantitative variables and all qualitative variables in a cluster are correlated (or anti-correlated) or the correlation ratios are equal to 1, the homogeneity criterion is maximized. The second aspect focuses on the definition of a synthetic variable of each cluster by a principal component approach for mixed data. Afterwards, the values of the synthetic variables are used via AHC to cluster the herds (Chavent et al., 2012; Brida et al., 2014).

### 2.3 Comparison of herd clusters for cow traits

In this regard, due to the largest ASW, we considered the four HCs to be created by the CoVAHC application (details are presented in Sect. 3.1). Cow traits were from the recording years 2017 and 2018. The number of cows per HC was as follows: HC1 of 1091 cows, HC2 of 64 cows, HC3 of 1059 cows and HC4 of 3324 cows. Production data considered 55 181 repeated test-day records from 5538 cows (19 964 records from 1947 DSN cows and 35 217 records from 3591 HF cows) for milk yield (Mkg), protein yield (Pkg), fat yield (Fkg) protein percentage (P%), fat percentage (F%), somatic cell sore (SCS) and fat-to-protein (FPR) ratio from the first to the third lactations. Female fertility traits included the interval from calving to first insemination (CFI) and the success of a first insemination (SFI). In this regard, we considered 6100 observations for CFI from 4562 cows (2548 records form 1871 DSN cows, and 3552 records from 2691 HF cows) and 7333 first inseminations for SFI from 5119 cows (3119 records form 2096 DSN cows, and 4214 records from 3023 HF cows). The udder health indicator somatic cell count (SCC) was log transformed into SCS = log2 (SCC / 100 000 cells) + 3 (Ali and Shook, 1980).

Linear mixed models were applied to assess the effect of defined HC on the cow test-day traits: Mkg, Pkg, Fkg, P%, F%, SCS and FPR. All calculations were performed with R version 4.0.2 (R Core Team, 2020) and applied the package “emmeans” (Lenth, 2020). This package was also used to calculate least squares means (LSMs) for traits within HCs and to test for corresponding significant differences. The respective model (model 1) was defined as follows:

1 $$\begin{array}{rl} y_{ijklmno} & =\mu +B_{i}+{\mathrm{YS}}_{j}+L_{k}+{\mathrm{HC}}_{l}+{\mathrm{DIM}}_{m}+{\mathrm{CA}}_{n}\\ & +A_{o}+e_{ijklmno}, \end{array}$$

where μ is the overall mean effect, and the fixed effects are as follows: B is the breed (DSN or HF), YS is the year season of calving (December–February, March–May, June–August or September–November), L is the lactation number (1, 2 or 3), HC is the herd cluster (HC1, HC2, HC3 or HC4), DIM is the fixed regression on days in milk (Legendre polynomials of third order), and CA is the calving age as covariate (linear regression), A is the animal as a random effect, and e is the random residual effect.

A linear mixed model was applied to CFI, and a generalized linear mixed model with a logit-link function was applied to SFI. Effects in the respective model (model 2) were the same for both female fertility traits and defined as follows:

2 $$\begin{array}{rl} y_{ijklmnop} & =\mu +B_{i}+{\mathrm{MI}}_{j}+L_{k}+{\mathrm{HC}}_{l}+{\mathrm{SE}}_{m}+{\mathrm{CA}}_{n}\\ & +A_{o}+S_{p}+e_{ijklmnop}, \end{array}$$

where μ is the overall mean effect, and the fixed effects are as follows: B is the breed (DSN or HF), MI is the month of insemination (January–December), L is the lactation number, HC is the herd cluster, SE is the type of semen (fresh semen, deep frozen semen, natural mating), and CA is the age at insemination as covariate (linear regression); as random effects, A is the animal and S is the service sire, and e is the random residual effect.

### 2.4 Genetic evaluation models

Genetic evaluations for test-day milk yield considered phenotypic data from calving years 2012 to 2018 from the 5538 cows with 55 181 test-day records (35 217 records from DSN and 19 964 records from HF) from the first three lactations. The estimation of genetic parameters and breeding values was carried out for a test-day model (model 3) with a herd-test-day (HTD) or herd-cluster-test-day (HCTD) effect and for an alternative test-month model (model 4) with a herd-test-month (HTM) or herd-cluster-test-month (HCTM) effect. Again, we considered the four HCs from the CoVAHC approach. For the genetic parameter estimations with the DMU software package (Madsen and Jensen, 2013), the following linear animal models were defined:

3yijklmnop=μ+Bi+YSj+LAk+HTDlorHCTDl+DIMm+CAn+Ano+PEp+eijklmnop4yijklmnop=μ+Bi+YSj+LAk+HTMlorHCTMl+DIMm+CAn+Ano+PEp+eijklmnop,

where $y_{ijklmnop}$ is the test-day milk yield, μ is the overall mean; $B_{i}$ is the fixed breed effect; YS${}_{j}$ is the fixed year season of calving effect (1–27); LA${}_{k}$ is the fixed lactation effect (1, 2, 3); HTD${}_{l}$ or HCTD${}_{l}$ is the fixed effect of herd test day or herd-cluster test day; HTM${}_{l}$ or HCTM${}_{l}$ is the fixed effect of herd test month or herd-cluster test month; DIM${}_{m}$ is the fixed regression on days in milk using Legendre polynomials of third order; CA${}_{n}$ is the calving age as covariate (linear regression); An${}_{o}$ is the random additive genetic effect; PE${}_{p}$ is the random permanent environmental effect; $e_{ijklmnop}$ is the random residual effect.

## 3 Results and discussion

### 3.1 Evaluation of herd clustering approaches

**Figure 1:**
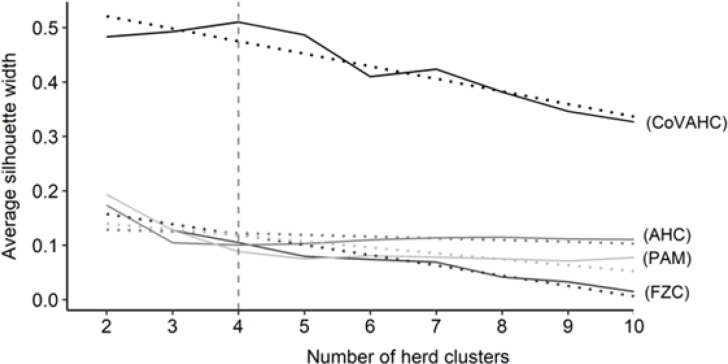
Average silhouette width for the different numbers of herd clusters considering the following clustering approaches: agglomerative hierarchical clustering (AHC), partition around medoids (PAM), fuzzy clustering (FZC), clustering of variables combined with agglomerative hierarchical clustering (CoVAHC); dotted lines are smoothed conditional means; the vertical dotted line indicates the chosen number of herd clusters for ongoing studies.

The first step was to determine the optimal number of HCs. Figure 1 shows the ASW for the four clustering methods, indicating a wide range from 0.015 (FZC with 10 HC) to 0.510 (CoVAHC with four HCs). Such huge variation for ASW displays the differences in separation efficiency of the different approaches. The ASW was highest (0.510) when creating four HCs and applying CoVAHC clustering. Such desired value for ASW for selected clustering procedures is in agreement with Gorgulu (2010), Ivemeyer et al. (2017) and Guiomar et al. (2018). For the remaining clustering approaches AHC, PAM and FZC, the ASW was quite stable in dependency of HC variations, but generally, ASW obviously declined for more than four HCs (Fig. 1).

In the Supplement (Fig. S1), ASWs for individual herds are shown. The different herd numbers as presented on the y axis are consistent for all clustering approaches and followed the herd numbering from the CoVAHC approach. The same design and pattern of bars represent the best overlap of herds in relation to the HCs as created by CoVAHC. Nevertheless, differences in herd separation accuracy among the clustering methods are very obvious. The CoVAHC application implied the largest silhouette width for all herds apart from herd 2, due to the missing contemporary herds in HC3. Individual herds displaying a negative silhouette do not reflect the characteristics of the remaining herds from the same HC, indicating misclassifications. Such misclassifications are very obvious for AHC, with negative silhouettes for the entire HC3 and herd 25. PAM displayed fewer misclassifications (only for herds 1, 11 and 12). With regard to FZC, only herd 12 indicated an incorrect HC assignment. Nevertheless, the ASW from the FZC approach indicated herd allocation inferiority compared to CoVAHC.

The evaluations of HCs in the present study focused on aspects with relevance for data recording and for genetic evaluations. The collected herd characteristics in this study represented different types of data, i.e., qualitative or quantitative. In order to overcome such obstacles, previous studies (Toro-Mujica et al., 2012; Riveiro et al., 2013; Ivemeyer et al., 2017; Blanco-Penedo et al., 2019) applied principal component analysis (PCA) to translate the categorical data structure indirectly into quantitative variables. These studies suggested a PCA due to the pronounced variation as identified among the most important principal components. To prevent possible biases through indirect transformations, Struyf et al. (1996) suggested a modified Gower distance. As a further method for handling mixed data types, Chavent et al. (2012) applied CoVAHC. They favored this approach over PCA, because more information can be taken into account when clustering the elements (herds). Furthermore, in contrast to PCA, orthogonality of the principal components is not required (Kuentz-Simonet et al., 2017).

### 3.2 Description of herd clusters for the optimal clustering approach (CoVAHC)

**Figure 2:**
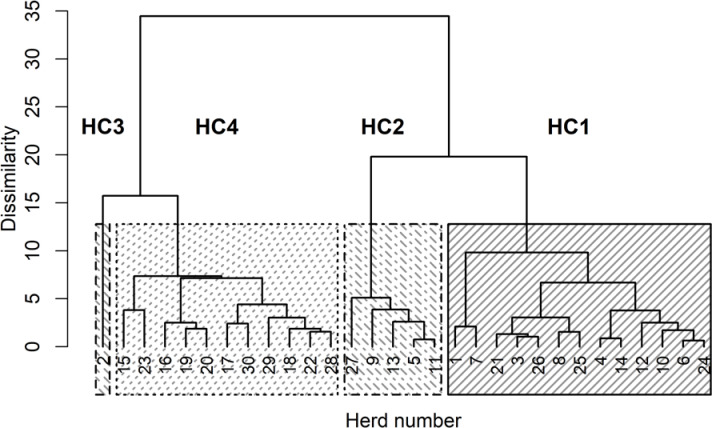
Dendrogram of the herds merged to herd clusters by clustering of variables combined with agglomerative hierarchical clustering (CoVAHC). Each block with specific pattern represents one HC.

The four HCs formed by CoVAHC, which are shown in Fig. 2, differ in multiple farm characteristics (Table 2), which in turn were used to describe the HC. The two breeds (DSN and HF) were clearly separated, meaning that HF herds only appeared in HC4. Overall, 91 % of herds from HC4 represented HF, and only 9 % were DSN herds. The percentage of DSN herds in HC1, HC2 and HC3 was 100 %. Such herd allocation based on herd characteristics indicates that the evolutionarily closely related DSN and HF breeds (Biedermann et al., 2005) are kept in different production systems representing a different herd management. The DSN are mostly kept in low input or grassland systems (Jaeger et al., 2018), but HF mostly in free-stall farms applying all available modern management instruments especially with regard to feeding strategies (e.g., feeding of total mixed rations) (König et al., 2005). Accordingly, Ivemeyer et al. (2017) clearly separated HF from local breeds with small population size such as original Angler cattle or DSN. Tremblay et al. (2016) only considered herds in automatic milking systems. Despite the same milking technology, they identified obvious differences in production pattern, feeding and management characteristics between small (Jersey, Guernsey, Ayrshire) and large populations (HF, brown Swiss).

**Table 2:** Percentages and values of main herd characteristics displaying significant${}^{1}$ herd cluster differences.

Variable	Answer option	Herd	Herd	Herd	Herd	Signi-
		cluster 1	cluster 2	cluster 3	cluster 4	ficance
		(n=13)	(n=5)	(n=1)	(n=11)	
Breed	DSN	100 %	100 %	100 %	9 %	${}^{***}$
	HF	0 %	0 %	0 %	91 %	
Herd size	median	51	15	800	145	${}^{***}$
Housing	cubicle stable	77 %	0 %	100 %	73 %	${}^{***}$
	compost systems	15 %	0 %	0 %	9 %	
	mixed systems	8 %	0 %	0 %	18 %	
	tie stable	0 %	100 %	0 %	0 %	
Pasture	yes	100 %	100 %	0 %	36 %	${}^{***}$
	no	0 %	0 %	100 %	64 %	
Herd management program	yes	31 %	0 %	100 %	100 %	${}^{***}$
	no	69 %	100 %	0 %	0 %	
Treatment	manual	85 %	100 %	0 %	0 %	${}^{***}$
documentation	herd management program	0 %	0 %	100 %	36 %	
	health project	15 %	0 %	0 %	64 %	
Feeding ration	maize focus	0 %	0 %	0 %	45 %	${}^{***}$
	grass focus	85 %	80 %	100 %	9 %	
	50 / 50 (maize–grass)	15 %	0 %	0 %	45 %	
	crude fiber	0 %	20	0 %	0 %	
Concentrated feed	mean (in kg)	3.50	1.30	8.50	6.10	${}^{**}$
Feed analyses	yes	31 %	20 %	100 %	82 %	${}^{*}$
	no	69 %	80 %	0 %	18 %	
Dry-off with antibiotics	yes	69 %	40 %	100 %	100 %	${}^{*}$
	no	31 %	60 %	0 %	0 %	
Natural service sire	bull: yes	77 %	60 %	0 %	18 %	${}^{***}$
	bull: no	23 %	40 %	100 %	82 %	
Artificial insemination by	farm personal	0 %	0 %	0 %	73 %	${}^{***}$
	inseminator	15 %	0 %	0 %	9 %	
	veterinarian	54 %	40 %	100 %	18 %	
	combined with bull	31 %	60 %	0 %	0 %	
Age of herd manager	mean (in years)	54.6	51.8	62.0	37.0	${}^{*}$
Agricultural experience	mean (in years)	35.0	37.2	50.0	19.9	${}^{*}$

${}^{1}$ Fisher's exact test was used for quality variables and Kruskal–Wallis test for quantitative variables to test for significant differences among the herd clusters (${}^{*}$ p<0.05, ${}^{**}$ p<0.01, ${}^{***}$ p<0.001).

It is interesting to note that both the HC1 and HC2 clusters included a mixture of conventional and organic herds, despite the substantial differences in legal regulations for both farming types. The organic farms from the present study base their feeding, breeding and management strategies on the guidelines for organic farming as defined by the European Union which are less strict than national German organic programs. Some particularities are defined in the basic principles for organic farming (IFOAM, 2020), especially addressing the breeding focus on longevity. Nevertheless, the allocation of organic as well as conventional herds to HC1 and HC2 suggests that there is an overlap of environmental conditions such as climatic impact between these two main classes (organic and conventional) affecting livestock production. Sorge et al. (2016) investigated herd management practices in organic and conventional dairy herds in Minnesota. They made similar conclusions, i.e., indicating that management decisions are diverse and herd specific, and do not strongly depend on the overall farming type organic or conventional. All of the HF herds as allocated to HC4 were conventional herds.

The majority of cows are kept in cubicle stables (HC1: 77 % of the herds, HC3: 100 % of the herds, HC4: 100 % of the herds) (Table 2). HC2 includes all herds with tie stables (16.7 % of all herds or 1.8 % of the cows), which usually have less than 20 cows. As identified for HC1, all cows in HC2 have access to pasture. In contrast, only one-third of the high-yielding herds (HC3 and HC4) reflect grazing systems. Accordingly, Müller-Lindenlauf et al. (2010) reported that herd productivity decreases with increasing length of the grazing period.

The cluster process (CoVAHC) separated the more traditional herds (herds in HC1 and HC2) from the more modern dairy herds (herds in HC3 and HC4). The level of digitization in animal housing, especially in dairy cattle farms, was defined as a major factor explaining herd and cow trait differences (Büscher, 2019). Herds allocated to HC2 did not use modern digital infrastructure (Table 2). Also, in HC1, the proportion of herds using a herd management software was comparatively low with 31 %. With regard to feeding strategies, herds from HC1 and HC2 use a very simple feed ration with only a few components, and they do not consider systematic feed analyses. In contrast, all herds in HC3 and HC4 base their management decisions on digital supporting systems. Also, the feeding rations are optimized considering scientific aspects and the needs of the cows. In total, 64 % of the herds from HC4 feed on a ration with a broad variety of ingredients.

The percentage of herds using natural service sires substantially differed among the HC (Table 2). The proportion was highest in HC1 with 77 %, followed by HC2 with 60 % but was quite low in HC4 (18 %). Herds from HC3 only considered artificial insemination. From a breeding perspective, Yin et al. (2014) identified utilization of natural service sires as a major characteristic when comparing organic with conventional farm types or DSN with HF herds.

With regard to social characteristics, mainly young farmers are responsible for the herd management in large-scale herds. Such a finding is in agreement with a comprehensive study across European dairy cattle herds (Blanco-Penedo et al., 2019). In HC4, the average age of herd managers was 37 years (Table 2). Nevertheless, the quite young farmers had substantial 20 years' experience in managing large-scale cow herds. The older farmers (average: 51.8 years with 37.2 years of agricultural experience) mainly managed the smaller herds (median: 15 cows) with tie stables (i.e., the herds from HC2).

### 3.3 Cow trait comparisons for the defined herd clusters

The comparison of LSM for test-day traits revealed significant differences (P<0.05; application of the Student's t test for pairwise differences) among the four HCs (Table 3). Hence, the different herd management strategies and farm characteristics as used for herd allocations simultaneously contributed to herd stratifications according to cow traits. A focus on milk production and high productivity was strongly associated with herd size. These herds were mostly assigned to HC3 (i.e., the “untypical DSN herd” with strong breeding focus on milk yield) and HC4 (i.e., the cluster including HF herds). Similar associations among herd variables and cow traits were reported by Müller-Lindenlauf et al. (2010), Ivemeyer et al. (2017) and Wallenbeck et al. (2018). HC2 represented herds with the lowest production level in Mkg (15.8 kg) and smallest herd sizes with a median of 15 cows. The mean production level of cows from HC1 was 21.2 kg milk, and the average herd size comprised 51 cows. The high-yielding HC3 and HC4 (HC3: 29.0 kg; HC4: 28.0 kg) represented the large-scale herds with 800 and 145 milking cows, respectively.

**Table 3:** Least square means and corresponding standard errors of test day and fertility traits in the first three lactations for four herd clusters created with CoVAHC.

	Trait	Unit	Herd cluster 1	Herd cluster 2	Herd cluster 3	Herd cluster 4
			(n = 13)	(n = 5)	(n = 1)	(n = 11)
Production	Milk	kg	21.16 ± 0.21${}^{b}$	15.78 ± 0.76${}^{a}$	29.01 ± 0.24${}^{d}$	28.04 ± 0.17${}^{c}$
	Protein	kg	0.74 ± 0.01${}^{b}$	0.57 ± 0.02${}^{a}$	1.05 ± 0.01${}^{d}$	0.96 ± 0.01${}^{c}$
		%	3.51 ± 0.01${}^{a}$	3.57 ± 0.03${}^{a}$	3.7 ± 0.01${}^{b}$	3.51 ± 0.01${}^{a}$
	Fat	kg	0.86 ± 0.01${}^{b}$	0.62 ± 0.03${}^{a}$	1.17 ± 0.01${}^{d}$	1.1 ± 0.01${}^{c}$
		%	4.21 ± 0.02${}^{b}$	4.11 ± 0.06${}^{\mathrm{ab}}$	4.11 ± 0.02${}^{a}$	4.05 ± 0.01${}^{a}$
	SCS	–	2.99 ± 0.04${}^{\mathrm{bc}}$	3.35 ± 0.15${}^{c}$	2.94 ± 0.05${}^{\mathrm{ab}}$	2.77 ± 0.03${}^{a}$
	FPR	–	1.21 ± 0${}^{c}$	1.16 ± 0.01${}^{b}$	1.11 ± 0${}^{a}$	1.16 ± 0${}^{b}$
Fertility	CFI	days	78.8 ± 6.99${}^{a}$	100.76 ± 7.67${}^{c}$	76.18 ± 7.16${}^{a}$	84.66 ± 7.05${}^{b}$
	SFI	–	0.67 ± 0.1${}^{c}$	0.66 ± 0.11${}^{\mathrm{bc}}$	0.53 ± 0.11${}^{\mathrm{ab}}$	0.45 ± 0.11${}^{a}$

Least square means in the same row with different superscripts a, b, c or d are statistically significant different at p<0.05, the lowest least square means value is indicated by ${}^{a}$ up to the highest value ${}^{d}$. SCS is the somatic cell score, FPR is the fat protein ratio, CFI is the time from calving to first insemination, and SFI is the success of a first insemination.

Cows from HC3 had the highest P% (3.7 %), but the remaining HCs did not differ significantly (P>0.05) for P% (Table 3). However, Pkg among HC differed significantly, which is due to the large differences in milk production. Similar observations were made for F% and Fkg.

HC2 comprised the herds with the highest SCS (average SCS: 3.35) (Table 3). In herds from HC2, all cows are housed in isolated tie stables with quite high air temperature and humidity. Inadequate hygiene and climate management contributed to impaired immune responses due to toxic gases (Barkema et al., 1999), resulting in increased SCS. Most of these herds (60 %) used an alternative dry-off management without antibiotic treatments. In contrast, the cows from the remaining HC are predominantly kept in loose housing and cold stalls, and the dry-off management is mostly based on antibiotic applications. The optimal climatic husbandry conditions plus preventive veterinary treatments might be an explanation for lower SCS in HC1 (2.99), HC3 (2.94) and HC4 (2.77) compared to HC2. Doherr et al. (2007) associated herd size with an increasing risk for clinical mastitis. In contrast, we identified lower SCS in HCs representing the large-scale herds. Doherr et al. (2007) and Ivemeyer et al. (2011) reported significant breed effects on SCS, indicating impaired udder health for HF cows. In our study, HC4 comprised all HF herds, and SCS in HC4 was lowest. Thus, the herd management and climatic conditions might have a stronger impact on the udder health status than herd size or breed (Barkema et al., 2015).

A FPR larger than 1.5 is an indicator for subclinical ketosis (Heuer et al., 1999). Surprisingly, the high productive DSN herd in HC3 considering large percentages of concentrates in the feeding ration is characterized by a significantly lower FPR (1.11) compared to the DSN herds from HC1 (1.21) and HC2 (1.16). The influence of the breed on FPR as described by Ivemeyer et al. (2019) was not confirmed in the present study, because the FPR in the “HF cluster” (HC4) was 1.16.

The LSM for SFI in HC1 (67 %) and HC2 (66 %) was significantly higher than in HC3 (53 %) and HC4 (45 %). The high proportion of natural matings in HC1 and HC2 (77 % and 60 %, respectively) explains such differences. Andersen-Ranberg et al. (2005) and Löf et al. (2012) reported a longer voluntary waiting period for a first insemination after calving in high-yielding herds. In our study, cows from HC1 and HC3 displayed the shortest CFI with 78.8 and 76.2 d, respectively, but both HC differed significantly with regard to milk yield (HC1: 15.8 kg vs. HC3: 29.0 kg).

### 3.4 Impact of herd clustering on genetic evaluations

Reliabilities of EBVs considering the whole population (cows and sires) and only for sires are given in Table 4. Statistical models including a herd-cluster-test-day or a herd-cluster-test-month effect increased the reliability in the entire population by 3.1 % and 3.5 %, respectively. Regarding sires, the increase was 5.1 % (herd-cluster test day) and 5.7 % (herd-cluster test month). Hence, the detailed consideration of environmental conditions and herd characteristics contributed to an increase of EBV reliabilities in the range from 3 % to 6 %, reflecting the postulations by Zwald et al. (2003) and Osorio-Avalos et al. (2015). The herd cluster instead of the herd effect for the CG modeling contributed to increased heritability estimates from 0.23 (herd-test-day effect and herd-test-month effect, respectively) to 0.36 (herd-cluster-test-day effect) or to 0.38 (herd-cluster-test-month effect).

**Table 4:** Heritabilities ($h^{2}$) with standard errors (SE) and reliabilities of estimated breeding values ($R^{2}$) with standard deviations (SD) for the test-day model with herd or herd-cluster effects and for the test-month model with herd or herd-cluster effect for the whole population and for sires with daughter records.

Model effect	Genetic	Whole population		Sires	
combined with	parameter	Herd	Herd	Herd	Herd
			cluster		cluster
Test-day	${h^{2}}_{\left(\mathrm{SE}\right)}$	0.253 (0.01)	0.378 (0.01)		
	${R^{2}}_{\left(\mathrm{SD}\right)}$	0.320 (0.28)	0.351 (0.31)	0.655 (0.13)	0.706 (0.13)
Test-month	${h^{2}}_{\left(\mathrm{SE}\right)}$	0.252 (0.01)	0.391 (0.01)		
	${R^{2}}_{\left(\mathrm{SD}\right)}$	0.320 (0.28)	0.355 (0.31)	0.655 (0.13)	0.712 (0.13)

The creation of herd-test-day effects in genetic evaluations for local breeds with small population size generally implies a limited number of records or animals in CG. This, in turn, can lead to biased genetic calculations (Strabel and Szwaczkowski, 1999). As an alternative, HC or HC-test-month effects were suggested as CG effects in genetic–statistical modeling approaches (Vasconcelos et al., 2008). The general aim of CG creation is to depict environmental conditions influencing cow traits as detailed as possible (Kuehn et al., 2007; Osorio-Avalos et al., 2015). In this regard, a clear differentiation among HCs is imperative, as realized when applying the CoVAHC clustering approach. CoVAHC generated four different HCs with obvious herd similarities within HCs, implying 496 different CGs in herd-cluster-test-day models (model 3). A classical herd-test-day modeling approach would generate 603 CGs, with only a few records for some effect levels. A model based on CoVAHC HC avoided the problem of weakly occupied effect levels. The minimal number was three records per CG when considering the herd-cluster-test-day effect in model 3. Consequently, a genetic evaluation based on CoVAHC HC will contribute to an increase in the effective number of daughters (Tosh and Wilton, 1994). In this regard, Schmitz (1990) addressed the positive impact on breeding value accuracies, especially for breeds with a small population size.

A final severe issue in genetic evaluations is the computation time, which is generally time consuming in classical test-day models with a large number of herd-test-day effects. In this regard, models 3 and 4 on a HC basis were superior over classical herd-test-day or herd-test-month models, with on average 5 %–10 % reductions in computation time.

## 4 Conclusions

The superiority of the CoVAHC approach over the AHC, PAM and FZC methods for herd clustering in the local DSN population could be clearly demonstrated. In this regard, German DSN herds were clearly allocated to different HCs based on broad spectra of social–ecological and herd characteristics. Hence, we postulate also correct herd groupings in other German cattle populations, when considering similar descriptors for herd characterization and when applying CoVAHC clustering. Other clustering methods as developed for other fields of science including AHC, PAM and FZC are not appropriate (due to the obvious herd misclassifications) for animal breeding objectives. Utilization of herd clusters instead of single herds is suggested in genetic evaluations for breeds with a small population size kept in small-sized herds with a limited number of contemporaries. The suggestion is based on the observed increased EBV reliabilities and heritabilities. The clustering approach for herd allocation with corresponding ongoing genetic evaluations is an alternative also for large-sized populations, when creating different feeding or management groups in the same herd.

## Supplement

10.5194/aab-64-187-2021-supplement The supplement related to this article is available online at: https://doi.org/10.5194/aab-64-187-2021-supplement.

## Data Availability

The data that support the findings of this study are available from the authors upon reasonable request.
