# Supplementary material for: Herd clustering strategies and corresponding genetic evaluations based on social–ecological characteristics for a local endangered cattle breed
Source: Arch Anim Breed. 2021 May 26;64(1):187–98. doi: 10.5194/aab-64-187-2021 (PMC8182665; doi:10.5194/aab-64-187-2021)
Supplement: The supplement related to this article is available online at: https://doi.org/10.5194/aab-64-187-2021-supplement. [file aab-64-187-supplement.zip › aab-64-187-2021-supplement-title-page.pdf]

Supplement of Arch. Anim. Breed., 64, 187–198, 2021
https://doi.org/10.5194/aab-64-187-2021-supplement
© Author(s) 2021. CC BY 4.0 License.

Open Access
Archives Animal Breeding

Supplement of

Herd clustering strategies and corresponding genetic evaluations based on social–ecological characteristics for a local endangered cattle breed

Jonas Herold et al.

Correspondence to: Sven König (sven.koenig@agrar.uni-giessen.de)

- aab-64-187-2021-supplement-title-page.pdf
- supplementary_Tables_Figures
  - Table_S1_Figure_S1.docx

The copyright of individual parts of the supplement might differ from the article licence.
