# Supplementary material for: Herd clustering strategies and corresponding genetic evaluations based on social–ecological characteristics for a local endangered cattle breed
Source: Arch Anim Breed. 2021 May 26;64(1):187–98. doi: 10.5194/aab-64-187-2021 (PMC8182665; doi:10.5194/aab-64-187-2021)
Supplement: The supplement related to this article is available online at: https://doi.org/10.5194/aab-64-187-2021-supplement. [file aab-64-187-supplement.zip › supplementary_Tables_Figures/Table_S1_Figure_S1.docx]

**Supplement**

**Table S1:** Survey for the social-ecological farm characterization and dairy cow herd description

| **Variable** | **Answer option** | **Variable** | **Answer option** |
| --- | --- | --- | --- |
| *General farm information* | | *Dry cow management* | |
| Breed | DSN  HF | Transit group | Yes/no |
|  |  | Special feeding of transit group | Yes/no  Yes, like dairy cattle |
| Herd size | [Numeric] |  |  |
| Legal form | Family farm  Company under civil law  Company with limited liability  Cooperative farm | Dry off time | [Numeric] |
|  |  | Dry off- antibiotic treatment | Yes/no |
|  |  | Dry off- homeopathic treatment | Yes/no |
|  |  | Non-antibiotic intramammary seal | Yes/no |
| Farm type | Conventional  Organic (EU)  Organic (organization) | Microbiological testing (of milk samples) at dry-off | Yes/no  Yes, if necessary  Yes, before antibiotic treatment |
| Types of production | Dairy farm  Livestock + crop farming | Calving place | In herd/ on pasture  Separate single calving box  Separate group calving box |
| Main income | Milk  Other |  |  |
|  |  | Cleaning interval of Calving pen | No, direct cleaning (more than 4 cows in a row in one box  After each calving, cleaning and disinfection  Re-bedding after each calving |
| Average altitude | [Numeric] |  |  |
| Average annual temperature | [Numeric] |  |  |
| Average annual rainfall | [Numeric] | Milk fever prophylaxis | Yes, if necessary  Yes, generally from 3rd lactation  No |
| Average Soil value number | [Numeric] |  |  |
| Soil type | [Numeric] |  |  |
| Terrain type | Flat  Hilly  Mountainous | *Calf management* | |
|  |  | Kind of calves‘ milk diet | Whole milk  Milk replacers |
| Leading soil associations | After geoviewer.de | Frequency of calf feeding | Ad libitum  Twice  Three times |
| *Housing system dairy cows* | |  |  |
| Housing | Free stall  Deep litter or compost system  Mix  Tie-stalls |  |  |
|  |  | Amount of milk per meal | [Numeric] |
|  |  | Calf loss | [Numeric] |
|  |  | *Heifer management* | |
| Housing climate | Cold  Warm  Mixed | Rearing of heifers | Own rearing  Extern |
|  |  | Average Heifer group size | [Numeric] |
| Stable flooring | Slatted floor  Slatted floor with rubber pad  Solid floors  Solid floors with rubber pad  Deep litter | Vaccination programme | [Numeric] |
|  |  | Heifer integration | No  One Week before Calving date  More than one Week before Calving date |
|  |  | Housing climate | See "Housing system dairy cows- Housing climate" |
| Cubicles | High cubicles  Deep cubicles  Deep litter or compost system  Tie-stalls | Housing | See "Housing system dairy cows- Housing" |
|  |  | Ground in barn | See "Housing system dairy cows- Ground in barn" |
|  |  | Cubicles | See "Housing system dairy cows- Cubicles" |
|  |  | Cubicles filling | See "Housing system dairy cows- Cubicles filling" |
| Cubicles filling | Rubber mat  Straw, lime-straw mix  Separated slurry  No cubicles | *Fertility management* | |
|  |  | Natural service | Yes/no |
|  |  | Artificial insemination by | Farm staff  Inseminator  Veterinarian  Combined with bull |
| Number of cubicles | [Numeric] |  |  |
| Number of feeding place | [Numeric] |  |  |
| Animal: cubical ratio | [Numeric: numeric] | Kind of heat control | Visual  Visual + technical support  Bull |
| Animal: feeding place ratio | [Numeric: numeric] |  |  |
| Overcrowding | Rarely  Yes  Never |  |  |
|  |  | Visual heat control | Yes/no |
|  |  | Separate time for heat control | Yes  No, as it fits into workflow |
| Cow brush | Yes/no |  |  |
| Open air outlet | Yes/no | Technical support for heat control | Yes/no |
| Rubber mat at walking alley | Yes/no | Breeding decisions | No advise  Breeding advisor  Breeding program |
| *Pasture management* | |  |  |
| Pasture | Yes/no |  |  |
| Kind of Pasture | No pasture  Strip grazing  Permanent pasture  Short-rotation pasture  Rotational grazing | Main breeding aim | Milk yield  Milk quality  Health  Body characteristics  Behaviour  Other |
| Pasture maintenance | Yes/no |  |  |
| Pasture access (yearly) | [Numeric] | Second breeding aim | See "Fertility management- Main breeding aim" |
| Pasture access (daily) | [Numeric] | Calving interval | [Numeric] |
| *Health management* | | *Daily workflow* | |
| Herd management program | Yes/no | Feeding Time | Before milking time  After the milking time  During milking  At separate times |
| Treatment documentation | Manual  Herd management program  Health project |  |  |
| Maternity vaccination | Yes/no | Adherence to the daily rhythm | Yes, very accurate  Yes, predominantly  Adapted to the workflow |
| Claw care | Farm staff  Extern  Farm staff + extern |  |  |
|  |  | Total employees | [Numeric] |
| Frequency of claw care per year | [Numeric] | Employees for dairy cattle | [Numeric] |
| Somatic cell count | [Numeric] | *1st Farm manager* | |
| Main culling reason | Milk yield  Health  Udder health  Claw problems  Other | Age of herd manager | [Numeric] |
|  |  | Family status | Single  Married  Divorced  Registered civil partnership |
| Second culling reason | See "main culling reason" | Number of children | [Numeric] |
| Frequency of displaced abomasum increases | [Numeric] | Care intensity of children | None  Light  Medium  High |
| Frequency of ketosis | [Numeric] |  |  |
| Frequency of acidosis | [Numeric] |  |  |
| Frequency of dermatitis digitalis | [Numeric] |  |  |
| Frequency of sole ulcer | [Numeric] | School education | Basic school qualification  Intermediate school-leaving certificate  Vocational baccalaureate diploma  General qualification for university entrance |
| Frequency of mastitis | [Numeric] |  |  |
| *Feeding dairy cows* | |  |  |
| Number of feeding groups | [Numeric] |  |  |
| Type of ration | Partial mixed ration  Total mixed ration  Only grass silage  Separate feed | Vocational training | No vocation training/ lateral entry  Agricultural training  Training as a technician  Master craftsman training  Studies of agricultural sciences |
| Ration | Maize emphasized  Grass emphasized  Fifty-fifty (maize- grass)  Crude fibre |  |  |
|  |  | Agricultural experience | [Numeric] |
|  |  | Time in the Farm | [Numeric] |
|  |  | Measures to improve herd management | Low  Medium  High |
| Average annual herd milk yield | [Numeric] |  |  |
| Average amount of concentrated feed per day | [Numeric] |  |  |
| Regular feed analyses | Yes/no | *2nd Farm manager* | |
| Ration complexity | Yes/no | Second Herd manager | Yes/no |
| Feeding frequency | [Numeric] | Age of herd manager | [Numeric] |
| Frequency pushing feed back to fence | [Numeric] | Family status | See "1st Farm manager - Family status" |
| Feeding dry cows | | Number of children | [Numeric] |
| Type of ration | See "Feeding dairy cows- type of ration" | Care intensity of children | See "1st Farm manager - Care intensity of children" |
| Ration | See "Feeding dairy cows- Ration" | School education | See "1st Farm manager - School education" |
| Average concentrated feed | [Numeric] | Vocational training | See "1st Farm manager - Vocational training " |
| Feed analyses | Yes/no | Agricultural experience | [Numeric] |
| Feeding frequency | [Numeric] | Time in the Farm | [Numeric] |
| Frequency pushing feed back to fence | [Numeric] | Measures to improve herd management | See "1st Farm manager - Measures to improve herd management" |


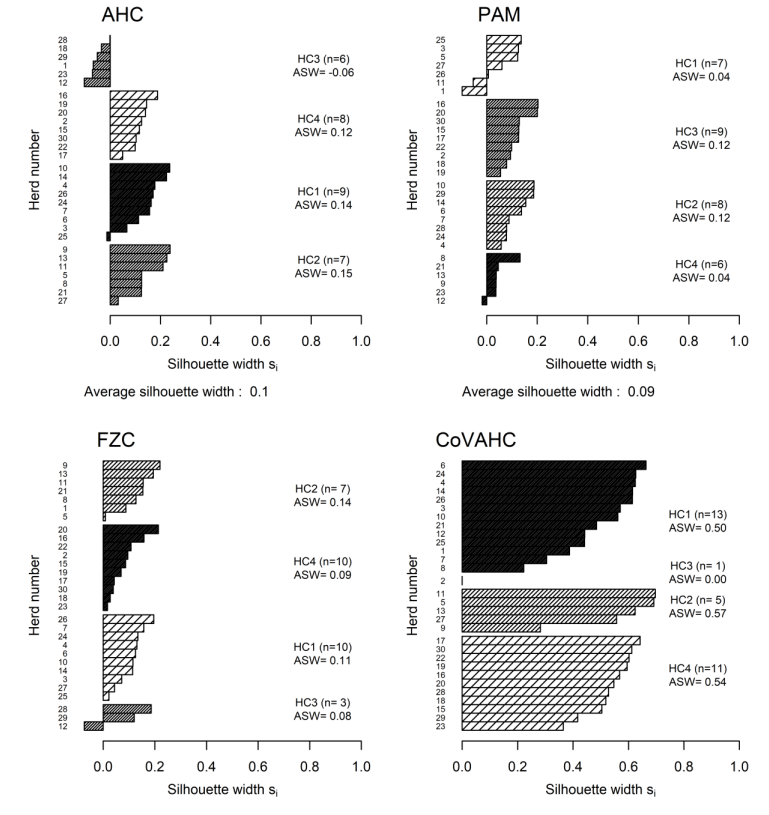


**Figure S1.** Average silhouette width (ASW) within herds and herd clusters (HC) when creating four HC with the clustering approaches agglomerative hierarchical clustering (AHC), partition around medoids (PAM), fuzzy clustering (FZC), clustering of variables combined with agglomerative hierarchical clustering (CoVAHC). Herd numbers are the same for all clustering approaches. The same design and pattern of bars represent the best overlap of herds in relation to the HC as created by CoVAHC.
